# NAD ^+^ -dependent Formate Dehydrogenase from Plants

**Published:** 2011

**Authors:** A.A. Alekseeva, S.S. Savin, V.I. Tishkov

**Affiliations:** Chemistry Department, Lomonosov Moscow State University; Innovations and High Technologies MSU Ltd; Bach Institute of Biochemistry, Russian Academy of Sciences

**Keywords:** plant formate dehydrogenase, physiological role, properties, structure; expression;*Escherichia coli*, protein engineering

## Abstract

NAD^+^-dependent formate dehydrogenase (FDH, EC 1.2.1.2) widely occurs in nature. FDH consists of two identical subunits and contains neither prosthetic groups nor metal ions. This type of FDH was found in different microorganisms (including pathogenic ones), such as bacteria, yeasts, fungi, and plants. As opposed to microbiological FDHs functioning in cytoplasm, plant FDHs localize in mitochondria. Formate dehydrogenase activity was first discovered as early as in 1921 in plant; however, until the past decade FDHs from plants had been considerably less studied than the enzymes from microorganisms. This review summarizes the recent results on studying the physiological role, properties, structure, and protein engineering of plant formate dehydrogenases.

## INTRODUCTION

NAD ^+^ -dependent formate dehydrogenases (FDHs) [EC 1.2.1.^2^] belong to the family of enzymes catalyzing the oxidation of the formate ion to carbon dioxide, coupled with NAD ^+^ reduction to NADH: 

HCOO ^-^ + NAD ^+ ^ → CO _2_ ↑ + NADH. 

It is possible to distinguish two major FDH groups based on the differences in structure of these enzymes. The first group is comprised of formate dehydrogenases from anaerobic microorganisms and archae. FDHs in this group are heterooligomers with a complex quaternary structure and a high molecular weight. They are generally characterized by the presence of various prosthetic groups (iron–sulphur clusters, molybdenum and tungsten ions) in the active site and high sensitivity to oxygen [1, 2].

The second group is comprised of NAD ^+^ -dependent formate dehydrogenases consisting of two identical subunits, both having two active sites and containing neither metal ions nor prosthetic groups in the protein globule. FDHs of this group belong to the superfamily of D-specific dehydrogenases of 2-oxyacids [3]. The reaction of formate oxidation catalyzed by a FDH from this group is the simplest example of dehydrogenation of carbonyl compounds, since there is neither the stage of proton transfer in the catalytic mechanism nor other stages of acid–base catalysis. The reaction rate is generally limited by the rate of hydride ion transfer from the substrate to the C4 atom of the nicotinamide ring [4]. Thus, FDH can be used as a model enzyme for studying the mechanism of hydride ion transfer in the active site of dehydrogenases that belong to this superfamily. 

The active and systematic study of FDHs began in the early 1970s and was primarily devoted to enzymes from microorganisms. The physiological role of microbial FDHs is different. Thus, in methanol-utilizing bacteria and yeast, this enzyme participates in the supply of energy to a cell, whereas in pathogenic bacteria and fungi FDH is a stress protein. The properties and protein engineering of FDH were thoroughly discussed in [5, 6].

NAD ^+^ -dependent formate dehydrogenases from plants also belong to the second FDH group. Recent studies have revealed that FDH also belongs to stress proteins in plants, similar to those found in pathogenic microorganisms. FDH synthesis strongly increases under the following conditions: drought, with abrupt changes in temperature, irradiation with hard ultraviolet light, through the action of chemical agents [7–9], hypoxia [10], and action of pathogenic microorganisms [11]. The significance of the physiological role of this enzyme gives rise to the necessity of studying plant FDHs. Till now, there are no publications in which the data on plant FDHs are systematized. The major features of plant formate dehydrogenases, as well as their kinetic properties and stability, are summarized in this review; a detailed description of the physiological role of FDH is also presented. 

## DISCOVERY HISTORY, LOCALIZATION,  AND PHYSIOLOGICAL ROLE OF PLANT FDHs

Plant FDH was first found in beans ( *Phaseolus vulgaris* ) in 1921 [12]. 

The first attempt to provide a detailed description of FDH and to assess the role of this enzyme in plant metabolism was made by Davison in 1951 [13], by the example of formate dehydrogenases from pea and bean seeds. The role of FDH was believed to consist in the production of NADH, which was subsequently consumed for the formation of ethanol, succinate, and glutamate in coupled reactions. Thus, the role of FDH as a “supplier” of NADH molecules to fit the various needs of a cell was first defined. An assumption concerning the mechanisms of the emergence of formate in a plant cell was made in the same study. According to the first hypothesis, formate could be formed along with ethanol and acetic acid as a result of anaerobic respiration. According to an alternative hypothesis, formate could be formed during the oxidation of glycolic acid; however, no unambiguous data that would corroborate a certain metabolic pathway, during which formate is formed, have been obtained thus far.

The first experiments for determining the localization of FDHs in plant cells were carried out in 1956. It was revealed that formate dehydrogenase activity was primarily present in mitochondria [14]. However, due to the fact that the samples under study were contaminated with other organelles, it couldn’t be unequivocally proven that FDH is localized in mitochondria. In 1960, it was demonstrated that FDH was localized not only in seeds, but also in other plant parts. Formate dehydrogenase activity was revealed in cabbage and spinach leaves, roots of garden radish and turnip, cauliflower buds, and pumpkin fruits [15]. It was demonstrated using spinach leaves that there were at least two pathways for formate oxidation in a plant cell: by FDH in mitochondria and by peroxidase in peroxisomes [16]. It was ascertained in separate experiments that formate was oxidized by FDH in mitochondria at pH > 6, while peroxidase in peroxisomes plays the major role in formate oxidation at lower pH values. Later, it was shown that FDH in mitochondria was a component of a protein complex with a molecular weight of approximately 200 kDa rather than being an individual molecule [17]. These complexes can potentially be formed by glycine decarboxylase and fumarase; their concentration increasing synchronically with rising FDH activity [9].

It was shown in systematic studies that formate dehydrogenase activity was strongly dependent both upon a plant and upon a particular plant organ containing the enzyme [18]. The dependence of enzymatic activity on the rate of oxygen consumption by a plant was also revealed. Thus, in plants with high oxygen consumption (spinach, tobacco, etc.), formate dehydrogenase activity was higher than that in a plant with low oxygen consumption (the Leguminosae, lettuce, etc.) [18]. In this study, the hypothesis was postulated that in oxidation of NADH obtained via the formate dehydrogenase reaction, the accumulated energy was consumed for ATP formation via the electron transport chain, thus satisfying the energy demand of the cell [18]. Unfortunately, high variation of FDH activity in different plants prevents the unambiguous answering of the question concerning the role of this enzyme in the metabolism. The relationship between formate metabolism and plant response to stress was first noted in 1978 [19], the increased formation of labelled carbon dioxide from formate was observed in barley, which was grown under overwatering conditions.

In 1992, research into the physiological role of formate dehydrogenase from plants was raised to a new level [20]. It was revealed that the mitochondria of non-photosynthesizing tissues of potato contained an unknown peptide with a molecular weight of approximately 40 kDa, which composed up to 9% of all mitochondrial proteins. cDNA of this polypeptide was cloned in 1993; the analysis of the amino acid sequence encoded by this cDNA demonstrated 55% homology with FDH from *Pseudomonas * sp. 101 [21]. Comparison of the N-terminal sequences of natural FDH and polypeptide translated from cDNA revealed that the theoretical protein contained an additional signal peptide consisting of 23 amino acid residues, which provided the transport of pro-enzyme from cytoplasm inside mitochondria. Polypeptides of the same molecular mass were found in pea, tomato, and onion; the FDH content in the mitochondria of non-photosynthetic tissues (tubers and roots) was approximately eightfold higher than that in leaves [20]. Moreover, FDH concentration sharply increased in plants which were grown in the dark (pea stems, chicory leaves, carrot roots, sweet potato tubers, etc.) [20]. 

Actually, numerous data have been published supporting the fact that FDH is synthesized at a high concentration under conditions that are unfavourable to plant growth, e.g.: drought, low temperature, hard ultraviolet radiation, exposure chemical agents, deficiency of both light and iron, and low oxygen concentration. However, the response rate strongly depended on the type of interaction. Thus, the fastest response of potato plants, which manifests itself by mRNA synthesis, was observed upon direct damage to plant tissue (~20 min), whereas the average response time for other types of impacts was equal to 8 h [7]. Under conditions of iron deficiency, the amount of formate dehydrogenase mRNA in barley roots began to increase after 1 day, attaining the maximum value after 14 days [8, 22], whereas the synthesis of formate dehydrogenase in leaves did not change. Under anaerobic stress, the concentration of FDH mRNA in barley roots increased as early as after 12 h, attaining the maximum value by the 48th hour. In maritime pine, the biosynthesis of FDH is enhanced during a drought [23]. An increase in the level of FDH mRNA was also observed in *Lotus japonicиs * plants cultivated under conditions of hypoxia [10]. 

The gene expression in moss *Physcomitrella patens * responding to stress was studied in [24]. Moss plants were treated with abscisic acid (hormone inducing the transfer of plants to the rest period and being capable of decelerating stem growth, which is accumulated in seeds and buds in Autumn) followed by cooling to +4 ^о^ С. It was found that abscisic acid induced an increase in resistance of moss to low temperatures; it also altered the set of expressed genes. FDH is one of the enzymes whose gene is expressed under the action of abscisic acid. It turned out that the level of FDH gene expression increased during several hours following treatment with abscisic acid and when the plants were stored in cold temperature for 24 h. In the absence of abscisic acid, the response to the impact of low temperatures occurs much more slowly. Treatment with sodium chloride at high concentrations (0.125 and 0.25 M) and mannitol (0.25 and 0.5 M) enhanced both the resistance of the moss to low temperatures and the expression of a number of genes, including the FDH gene. It was thus demonstrated that formate dehydrogenase was a stress protein both in higher plants and in mosses; the level of its biosynthesis could be regulated by hormones. Other plant hormones, such as auxin and cytokinin, also have an effect on FDH activity in higher plants [25]. 

The synthesis of FDH was also studied in *Arabidopsis thaliana,* being exposed to various factors. It was the first plant for which the complete nucleotide sequence of the genome was determined; therefore, in many cases *A. thaliana * is used as a model plant. The plants were sprayed with various C1-compounds (methanol, formaldehyde, and formate) followed by the Northern blot analysis using FDH cDNA as a probe. The most intensive expression of the FDH gene was observed for treatment with formaldehyde or methanol. A lower level of expression was observed in the samples sprayed with formate and deionized water. An increase in the expression of the FDH gene was not recorded, neither in plants with their leaves pruned nor in the control sample. These data enabled one to reasonably conclude that the synthesis of FDH was induced to a larger extent not by the formate substrate, but by its reduced form (formaldehyde) [26]. It was also demonstrated [27] that one-carbon compounds (methanol, formaldehyde, and formate) induced the synthesis of FDH in plant leaves. Methanol has a direct effect on the synthesis of FDH transcripts, while its oxidized modifications (formaldehyde, formate) can act as signalling molecules. An analysis of the N-terminal region of the enzyme allowed one to assume that FDH can also be transported to chloroplasts. The dual localization of FDH, both in mitochondria and chloroplasts, was shown in transgenic * A. thaliana* and tobacco plants containing the AthFDH gene [28]. 

The origin of formate in cells of the plants exposed to stress remains unknown. The hypothesis has been put forward suggesting that formate may be synthesized during photorespiration, in the methanol metabolism, or from glyoxylate formed from different products of the Krebs cycle [7]. The formation of formate by the serine pathway as it takes place in bacteria [1] has been discussed, since the introduction of serine resulted in the increase in FDH concentration in potato plants. In further experiments [29], the transgenic potato with suppressed synthesis of FDH was obtained. It was revealed that formate that does not undergo further oxidation to carbon dioxide was accumulated in the tissues of transgenic plants. It was also shown that proline and its precursor glutamate were formed at a high concentration in transgenic potato under conditions of drought.

The metabolism of formate and its physiological role have been well studied [30]. In photosynthesizing potato tissues, formate is the major precursor of all other carbon-containing compounds; it is basically synthesized via ferredoxin-dependent fixation of carbon dioxide. In other tissues, formate is a side product of photorespiration and some enzymatic processes; its formation seems to result from the direct reduction of carbon dioxide in chloroplasts. In potato plants, the metabolism of formate is associated with the synthesis of serine.

A close relationship between the biosynthesis of formate and serine also exists in *A. thaliana* [31]. Three lines of transgenic plants with enhanced expression of FDH were obtained. Formate concentration in transgenic plants was almost identical to that in wild-type *A. thaliana* . Following the introduction of labelled formate, the intensity of formation of radioactively labelled carbon dioxide in transgenic plants was much higher, whereas serine accumulation remained at the same level. Transgenic *A. thaliana* plants with an enhanced level of FDH gene expression were also obtained in [32]. 

Phosphorylation is the most important method of metabolism regulation. 14 proteins of potato mitochondria, which can be presented in the phosphorylated form, have been discovered [33]; among them FDH can also be found. The amino acid residues of mitochondrial FDH of potato, which undergo phosphorylation (Thr76 and Thr333) have been identified [34]. An analysis of the FDH structure demonstrated that these two threonine residues were located on the surface of a protein globule and could be easily accessible for kinases catalyzing the phosphorylation process. A high phosphorylation level is observed in the Е1-α subunit of pyruvate dehydrogenase (PDH). The phosphorylation of both FDH and pyruvate dehydrogenase is regulated by the variation of concentrations of NAD ^+^ , formate, and pyruvate, which attests to the similarity in the mechanisms of regulation of the function of these enzymes. The level of phosphorylation of the enzyme is considerably reduced with increasing concentrations of NAD ^+^ , formate, and pyruvate. It is assumed that pyruvate can be converted into formate in the reaction catalyzed by pyruvate formate lyase (PFL) followed by the oxidation of formate with the participation of FDH. 

Formate ion takes part in a great number of metabolic processes with complicated regulation, as can clearly be seen from the data provided. The most complete scheme of participation of formate in plant metabolism can be found in [11].

Recent studies have attested to the fact that FDH content in plant mitochondria increases in response not only to physical and chemical factors, but also as a result of a “biological attack”. The activation of biosynthesis of FDH was observed following infection of the English oak with the pathogenic fungus, *Piloderma croceum* [35]; wheat, with fungus *Blumeria graminis f. sp. tritici* [36]; and the common bean, with fungus *Colletotrichum lindemuthianum* [11]. The common bean genome contains three FDH genes; their expression is regulated by the type of exposure factor. It is assumed that synthesis of FDH in wheat is induced by methanol as a result of the impact of pectin methylesterase on pectin. In plants of the common tobacco *Nicotiana attenuate* damaged by *Manduca sexta* caterpillars, fatty acid conjugates initiating the synthesis of a number of proteins, including FDH, are released [37]. 

Summarizing this section, let us note that formate dehydrogenase is a universal enzyme involved in the cell stress response caused by both exogenic (negative ambient impact) and endogenic (deficiency of essential microelements, exposure to pathogens) processes. This fact attests to the key role of FDH in metabolism processes of higher plants. The production of mutant forms of FDH characterized by an enhanced catalytic activity and the insertion of their genes into the plant genome instead of wild-type enzyme genes represents a fundamentally new approach to the design of plants with an enhanced resistance to stress.

**Table 1:** The sources and contracted notations of formate dehydrogenases considered in the present study

Organism		FDH	Reference
Latin name	English name		
PLANTS			
*Antirrhinum majus*	Common Snapdragon	AmaFDH1	KEGG: EST 2545
*Aquilegia formosa x Aquilegia pubescens*	Buttercup	ApuFDH1	KEGG: EST 273
		ApuFDH2	[11]
*Arabidopsis thaliana*	Mouse-ear cress	AthFDH	EMBL AF208029
*Brachypodium distachyon*	Purple false brome	BdiFDH1	[11]
*Brassica napus*	Rapeseed	BnaFDH1	[11]
		BnaFDH2	KEGG: EST 21261
*Brassica oleracea*	Cabbage	BolFDH1	[11]
*Cryptomeria japonica*	Japanese cedar	CjaFDH1	KEGG: EST 5066
*Carica papaya*	Papaya	CpaFDH1	KEGG: EST 3924
*Citrus reticulata*	Tangerine	CreFDH3	KEGG: EST 11052
*Citrus sinensis*	Sweet orange	CsiFDH1	[11]
*Coffea canephora*	Coffea canephora	CcaFDH1	KEGG: EST 1007
*Festuca arundinacea*	Tall fescue	FarFDH1	KEGG: EST 5855
*Glycine max*	Soybean	SoyFDH1	GB AK244764, [38]
		SoyFDH2	GB Bt094321
		SoyFDH3	GB AK243932, [38]
		SoyFDH4	GB BT095613
		SoyFDH5	KEGG: EST 19520
*Gossypium arboreum*	Tree cotton	GarFDH1	KEGG: EST 1085
*Gossypium hirsutum*	Tree cotton	GhiFDH1	KEGG: EST 19680
*Gossypium raimondii*	Tree cotton	GraFDH1	KEGG: EST 213
*Helianthus annuus*	Common sunflower	HanFDH1	[11]
*Hordeum vulgare*	Barley	HvuFDH1	GB D88272, [8]
*Ipomoea batatas*	Sweet potato	IbaFDH	EMBL BM878811
*Lactuca saligna*	Willowleaf lettuce	LsaFDH1	KEGG: EST 1616
*Lotus japonicus*	–	LjaFDH1	GB FM865900, [10]
*Lycopersicon esculentum*	Tomato	LesFDH1	GB AJ849378
*Malus domestica*	Apple	MdoFDH	EMBL CN496368
*Manihot esculenta*	Cassava	MesFDH1	KEGG: EST 2788
*Medicago truncatula*	Barrel Medic	MtrFDH1	KEGG: EST 1503
*Mesembryanthemum crystallinum*	Common ice plant	McrFDH	GB BE035085
*Nicotiana tabacum*	Common tobacco	NtaFDH1	[11]
*Oryza sativa Japonica group*	Japanese rice	OsaFDH_Ja	GB AK065872, [39]
*Oryza sativa indica cultivar-group*	Indian rice	OsaFDH_In	GB CT832868, [40]
*Oryza sativa*	Rice	OsaFDH1	AB019533, [41]
*Panicum virgatum*	Switchgrass	PviFDH1	KEGG: EST 8602
*Phaseolus vulgaris*	Common bean	PvuFDH1	GB ACZ74695, [42]
*Phyllostachys edulis*	Moso bamboo	PedFDH	GB FP093692
*Physcomitrella patens*	Moss*Physcomitrella patens*	PpaFDH	GB XM001768721, [43]
*Picea glauca*	White spruce	PglFDH1	KEGG: EST 2327
*Picea sitchensis*	Sitka spruce	PsiFDH	GB EF085163, [44]
*Pinus pinaster*	Maritime pine	PpiFDH1	KEGG: EST 174
*Pinus taeda*	Loblolly pine	PtaFDH1	[11]
		PtaFDH2	KEGG: EST 2972
		PtaFDH3	KEGG: EST 15504
*Populus nigra*	Lombardy poplar	PniFDH2	KEGG: EST 7989
*Populus tremula*	Aspen	PtmFDH1	KEGG: EST 4757
*Populus trichocarpa*	Western balsam poplar	PtrFDH1	PtrFDH1 GB XM002320465, [45]
*Prunus persica*	Peach tree	PpeFDH1	KEGG: EST 4281
*Quercus robur*	English oak	QroFDH1	GB AJ577266.2, [35]
*Raphanus raphanistrum subsp. raphanistrum*	Wild radish	RraFDH1	KEGG: EST 15157
*Ricinus communis*	Castor bean tree	RcoFDH1	GB XM_002517292
*Saccharum officinarum*	Sugarcane	SofFDH1	KEGG: EST 18227
*Solanum tuberosum*	Potato	StuFDH1	GB Z21493, [21]
*Sorghum bicolor*	Sorghum	SbiFDH1	GB XM002438363, [46]
		SbiFDH2	GB XM002454363, [46]
*Taraxacum officinale*	Common dandelion	TofFDH1	[11]
*Theobroma cacao*	Cacao tree	TcaFDH1	KEGG: EST 10274
*Triphysaria pusilla*	Dwarf owl’s-clover	TpuFDH1	KEGG: EST 5550
*Triticum aestivum*	Common wheat	TaeFDH1	TaeFDH1 GB AK332605, [47]
*Vigna unguiculata*	Cowpea	VunFDH1	KEGG: EST 6491
*Vitis vinifera*	Grape	VviFDH1	GB XM002278408
*Yucca filamentosa*	Bear grass	YfiFDH1	YfiFDH1 [11]
*Zea mays*	Maize	ZmaFDH	GB EU967680, [48]
*Zingiber officinale*	Ginger	ZofFDH1	KEGG: EST 5316
fungi			
*Aspergillus oryzae*	Fungus*Aspergillus oryzae*	AorFDH1	NCBI XM001827498
*Mycosphaerella graminicola*	Fungus*Mycosphaerella graminicola*	MgrFDH	GB AW180713 180985
*Penicillium marneffei*	Fungus*Penicillium marneffei*	PmaFDH1	GB XM002153251
*Ajellomyces capsulatus*	Darling’s disease fungus	AjcFDH1	[49]
		AjcFDH3	
*YEASTS*			
*Candida boidinii*	Methylotrophic yeast*Candida boidinii*	CboFDH	EMBL AF004096
*Saccharomyces cerevisiae*	Baker’s yeast	SceFDH	EMBL Z75296
BACTERIA			
*Pseudomonas sp. 101*	Methylotrophic bacterium*Pseudomonas sp. *101	PseFDH	[50]
*Moraxella sp. С2*	Methylotrophic bacterium*Moraxella sp.*	MorFDH	EMBL Y13245
*Burkholderia stabilis*	Bacterium *Burkholderia stabilis*	BstFDH	[51]
*Bordetella bronchiseptica RB50 (Alcaligenes bronchisepticus)*	Bacterium *Bordetella bronchiseptica *RB50* (Alcaligenes bronchisepticus)*	BbrFDH	EMBL BX640441
*Uncultured marine alpha proteobacterium*	Uncultured marine alpha proteobacterium	BbrFDH	EMBL BX640441
*Sinorhizobium meliloti*	Nitrogen-fixing*Sinorhizobium meliloti*	SmeFDH	Ae006469, [52]

## THE FEATURES OF THE PRIMARY STRUCTURE OF PLANT FDH

**Fig. 1:**
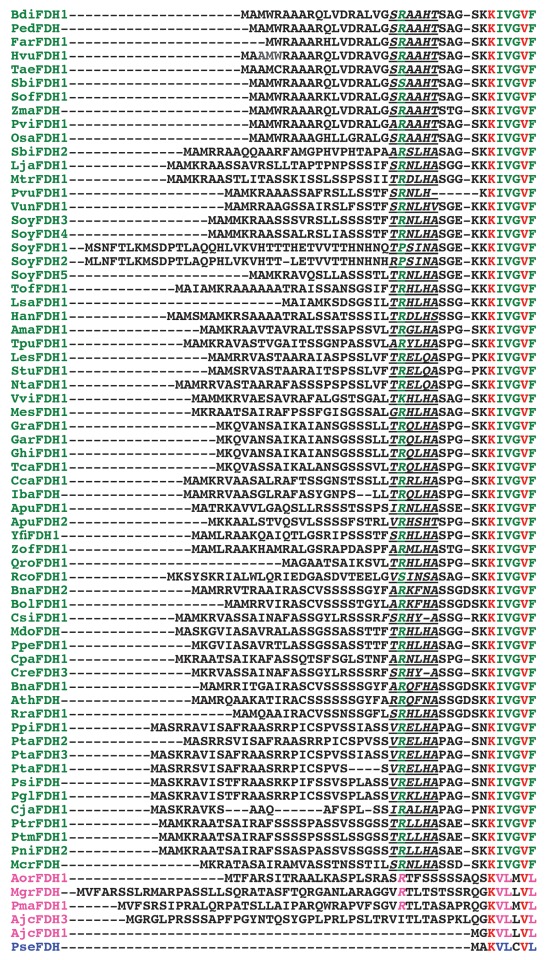
Signal sequences of plant formate dehydrogenases. Here and in *Figs. 2,3* , abbreviations of enzymes are those from *Table*   *1* . Plant enzymes are highlighted in green; fungi enzymes are highlighted in magenta; FDHs from bacteria are highlighted in blue. Specific sequences that are responsible for the transport of the enzyme to mitohondria are underlined. The residue, after which the signal peptide is eliminated, are highlighted in green italics.

Due to the active development of mega-sequencing methods, a new genome structure of various organisms, including plants, is published almost every day. Searching in GenBank (GB), EMBL, and KEGG (http://www.genome.jp/) databases enabled us to find nucleotide sequences of genes (complete or as cDNA) of plant FDH from over 70 sources. Moreover, study [11] presents a number of sequences that are not present in the databases. *Table 1* lists the names of the plants and the contracted notations of FDHs. FDHs that are characteristic of various microorganisms, such as enzymes from methylotrophic bacteria *Pseudomonas*  sp. 101 (the most well studied FDH to this moment), *Moraxella*  sp. C2, pathogenic bacteria *Burkholderia stabilis* and *Bordetella bronchiseptica RB50 (Alcaligenes bronchisepticus)* , uncultured marine alpha proteobacteria and nitrogen-fixing bacteria *Sinorhizobium meliloti* , yeasts *Saccharomyces cerevisiae* and *Candida boidinii* , were used for comparison. 

**Fig. 2:**
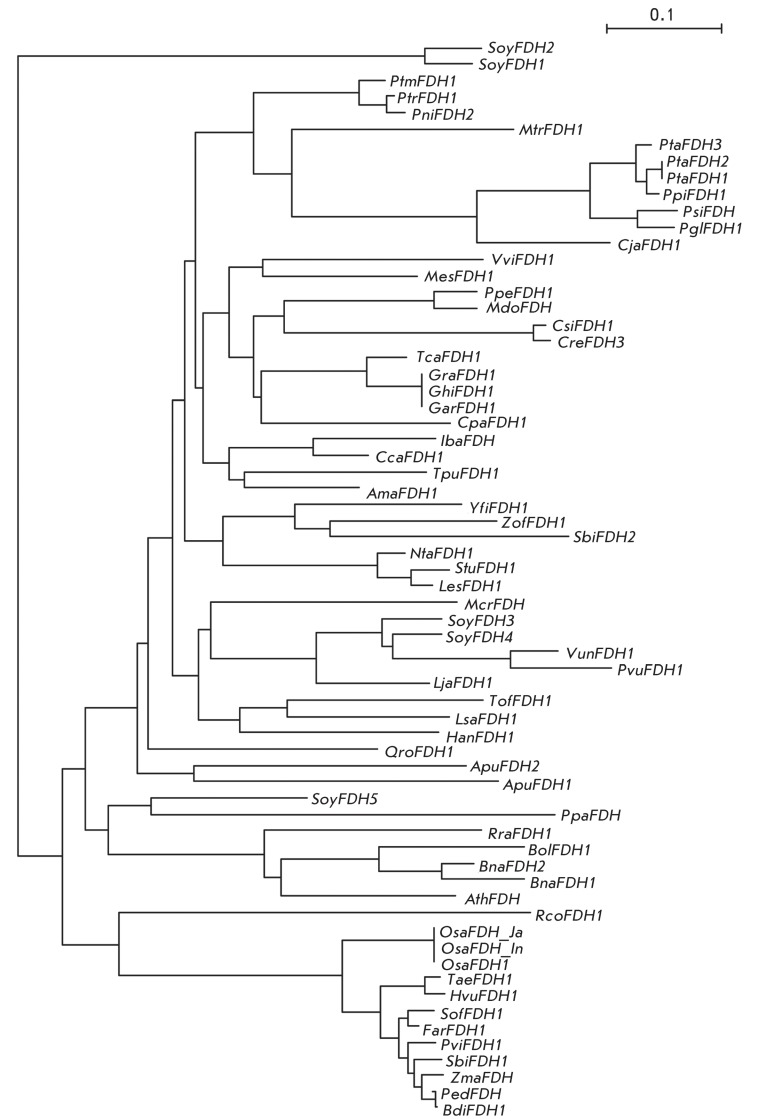
Phylogenetic tree of N-terminal sequences for plant formate dehydrogenases.

The presence of a signal peptide, which is responsible for FDH transport from the cytoplasm to mitochondria, at the N-terminus of the synthesized proenzyme is the distinctive feature of plant FDHs [21]. Bacterial and yeast FDHs contain no signal peptides. The genes of FDHs from a number of pathogenic fungi also contain the nucleotide sequence encoding the signal peptide. However, depending on the condition of a host cell, the RNA synthesized from the FDH gene undergoes alternative splicing, resulting in the formation of different mRNAs encoding proteins both with and without the signal peptide [49].

**Fig. 3:**
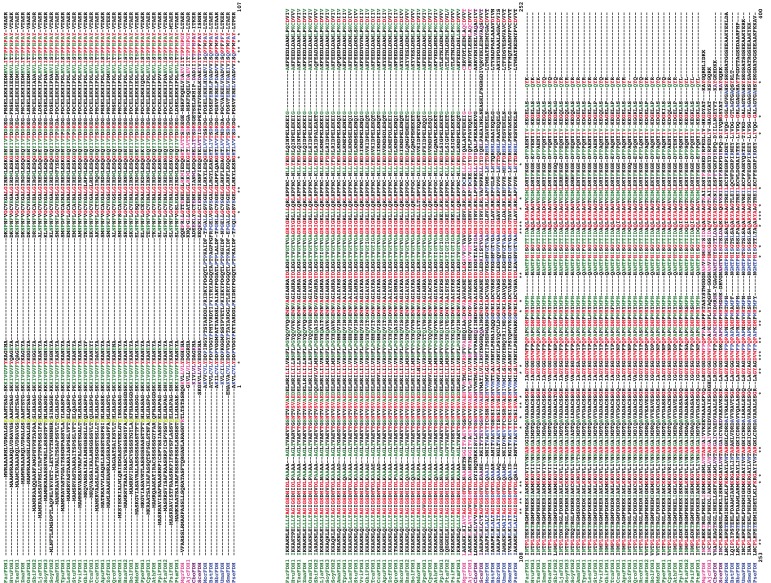
Alignment of formate dehyfrogenases from different sources. Abbreviations of enzymes see in *Table*  1. Plant enzymes are highlighted in green; fungi enzymes are highlighted in magenta; FDHs from bacteria are highlighted in blue. Specific sequences that are responsible for transport of the enzyme to mitochondria are underlined. The numeration of residues is the same as for the FDH from *Pseudomonas*  sp. 101 (PseFDH). The asterisk and red font mark the conserved amino acid residue.

*Fig. 1*shows the signal sequences of formate dehydrogenases from various sources. The potential specific sequences providing the transport of an enzyme to mitochondria are underlined. The residue, after which the cleavage of the signal peptide occurs, is shown in green italics. In the majority of formate dehydrogenases, it is the arginine residue. Serine residue (FDH from sorghum SbiFDH1, castor bean tree RcoFDH1), lysine (grape VviFDH1), proline (FDHs from soybean SoyFDH1 and SoyFDH2, isoforms 1 and 2) can also be found in this position. The signal sequence of FDH is enriched in amino acid residues containing hydroxyl or positively charged groups and is capable of forming an amphiphilic α-helix. The signal sequence is highly conserved. Thus, the deletion of only two N-terminal amino acids blocks the transport of the enzyme to the mitochondria [53]. It was ascertained that the N-terminal MAM motif enabled swift transport of the enzyme to mitochondria to be performed. *Fig. 1* shows the N-terminal sequences of 63 plant FDHs; 35 enzymes of those have the MAM motif at position 1–3. A number of plant FDHs have similar motifs at their N-terminal fragments: MAAM (in two plant FDHs) and MAS (in eight plant FDHs). The N-terminal amino acid sequences of isoenzymes 1 and 2 of soybean FDHs are significantly different from those of other plant formate dehydrogenases, both in terms of their composition (it starts with MSN an MLN) and size, which attests to the possible specific function of these FDH isoforms. The N-terminal sequences of FDHs from fungi *Aspergillus oryzae, As. flavus, Penicillium marneffei* , *Mycosphaerella graminicola, * and *Ajellomyces capsulatus* are also shown in *Fig. 1* (the names are highlighted in pink). Two enzyme isoforms from *Aj. capsulatus* (AjcFDH1 and AjcFDH3) are also given, which result from the alternative splicing of mRNA [49]. Some sequences also contain an arginine residue, at which the cleavage of the signal peptide may occur (the residue is highlighted in pink italics). A similar mechanism of the FDH transport to different cell organelles seems to exist in fungi. The Lys and Val residues that are totally conserved in all formate dehydrogenases are shown in red in *Fig. 1* . The N-terminal sequence of formate dehydrogenase from *Pseudomonas * sp. 101 that is highly homologous to the N-terminal region AjcFDH3 without the signal peptide is shown for comparative purposes. As can be seen in *Fig. 1* , signal peptide sequences in enzymes from the plants belonging to one family (the family Solanaceae: tomatoes, potato; the family Gramineae: rice, barley, rye, etc.) have a high degree of homology. 

In most plants, FDH is located in the mitochondria; however, the thorough study of the signal peptide of the enzyme from *A. thaliana* has shown that the enzyme can also be transported to chloroplasts. The N-terminal fragment of this enzyme strongly differs from the signal sequences of FDHs from potato, barley, and rice. There is a hypothesis that under certain conditions AthFDH localizing in chloroplasts is capable of catalyzing the reverse reaction, i.e., the conversion of carbon dioxide into formate [27]. It was shown by using another algorithm to compare the signal peptides of FDHs that all the enzymes, with the exception of FDH from tomato plants, were capable of being transported both to mitochondria and chloroplasts [28]. An analysis of signal sequences carried out using the Predotar, TargetP, and Mitoprot software [11] confirmed the fact that FDH is basically localized in mitochondria. 

In plants, formate dehydrogenase is often represented by several isoforms, also known as isoenzymes, whose synthesis is determined by the condition of a plant. The differences in the isoenzyme composition of FDHs in healthy and affected palms *Pericopsis mooniana* are used to select trees when selective cutting is performed [54]. Polymorphism is also typical for FDH from the almond tree *Prunus dulcis * [55] or *P. amygdalus* [56]. Based on the data of the analysis of isoforms of FDHs and several other dehydrogenases, a method for identifying plant genotype was proposed. As previously mentioned above, phosphorylation may be the reason for the formation of different FDH isoforms [34]. Depending on the modification degree, the formation of numerous forms of the enzyme with p *I* varying from 6.75 to 7.19 was observed. Moreover, it was ascertained that the additional isoforms of potato FDH emerged as a result of post-translational deamidation of Asn329 and Gln330 residues [34]. 

The differences in sequences of signal peptides are more strongly pronounced when compared with those in plant FDHs; the homology level between which is ~80%. It is most clearly demonstrated in the case of isoenzymes of soybean FDH. Homology of the sequences of isoenzymes is 98%, whereas for the signal sequences, it is lower than 40%. *Fig. 2* shows a phylogenetic tree of the signal peptides of plant FDHs. As can be seen, two isoenzymes of FDH from soybean *Glycine max* , SoyFDH1 and SoyFDH2, form an individual group. The N-terminal fragment of these enzymes is much longer than that in FDHs from other plants ( *Fig. 1* ) and strongly differs in terms of its amino acid composition. Next, there is a large branch of the family Gramineae, which includes the enzymes of rice (OsaFDH), wheat (TaeFDH), barley (HvuFDH), sugarcane, moso bamboo, etc. A large group is represented by the enzymes of plants belonging to the family Brassicaceae (Cruciferae), namely, wild radish (RraFDH), cabbage (BolFDH), rapeseed (BnaFDH), and *A. thaliana* (AthFDH). The other groups are formed of proteins from Asteroideae, Leguminosae, Solanaceae, Malvaceae, Pinaceae, and Salicaceae. As previously mentioned, five isoenzymes of soybean FDH do not form a separate group. Since the signal peptide is basically responsible for the transport of the enzyme inside the cell, an assumption can be made that different isoenzymes of soybean FDH are transported into different organelles. 

*Fig. 3* shows the alignment of some complete sequences of plant FDHs that are known at the present time; the sequences of a number of similar enzymes from microorganisms are provided for the purposes of comparison. The absolutely conserved regions are highlighted in red; the residues repeating only in plant FDHs are highlighted in green. A significant difference of bacterial enzymes from the plant ones is an N-terminal rigid loop. This region embraces a considerable part of the enzyme subunit. Its interaction with other amino acid residues is likely to be accounted for by a higher thermal stability of bacterial enzymes as compared with the plant ones. In addition, in FDHs of microbial origin, the C-terminal region is longer than that in plant enzymes. Meanwhile, the differentiation of FDH into two groups on the basis of its homology is clearly observed in the remaining part of the amino acid sequence. The first group contains bacterial and plant enzyme, whereas the second group consists of the enzymes from yeasts and fungi.

**Table 2:** Kinetic parameters of formate dehydrogenases from different sources

FDH specimen	Specific activity, U/mg	*k*_cat_, s^–1^	*K*_M_(NAD^+^), µM	*K*_M_ (formate), mM	*K*_M_(NADP^+^), mM		Reference
*Arabidopsis thaliana*, native, after affinity chromatography	NA*	NA	65	10	NA	NA	[27]
*A. thaliana*, native, from mitochondria	NA	NA	76	11	NA	NA	[59]
*A. thaliana*, recombinant from transgenic tobacco	1.3	0.87	78	11	NA	NA	[59]
*A. thaliana*, recombinant from transgenic tobacco + thermal treatment for 5 min at 60^o^C	0.1	0.07	35	3.3	ND	ND	[59]
*A. thaliana*, from mitochondria from leaves	1.9	1.27	34	1.4	NA	NA	[60]
*A. thaliana*, recombinant from*E. coli*	6.5	3.8	20	2.8	10	5.0 × 10^-5^	[58]
Pea (seeds) *Pisum sativum*	NA	NA	22	2 [61]1.67; 6.25 [62]	NA	NA	[61, 62]
Mung bean,*Phaseolus aureus, *native	NA	NA	7.2	1.6	NA	NA	[63]
Soybean,*G. max*, native	NA	NA	5.7	0.6	NA	NA	[57]
Soybean,*G. max*, recombinant	4.0	2.83 [64]	13.2	1.5	1	8.7 × 10^-4^	[58, 64, 76]
Lotus japonicus	NA	1.2 (NAD^+^)0.005 (NADP^+^)	29.5	6.1	29.5	3.7 × 10^-6^	[10]
Spinach,*Spinacia oleracea L.*, from leaves	NA	NA	NA	1.7	NA	NA	[16]
Potato *Solanum tuberosum*	NA	NA	19	0.54	NA	NA	[29]
*C. boidinii*,wild-type	6.3	3.7	37	5.9	NA	NA	[65]
*C. metillica, *wild-type	2.1	1.4	55	NA	NA	< 4 × 10^-6^	[66]
*Saccharomyces cerevisiae*, recombinant	10	6.5	36	5.5	NA	< 3.3 × 10^-10^	[67, 68]
Burkholderia stabilis	NA	1.66 (NAD^+^)4.75 (NADP^+^)	1430	55.5	0.16	25.0	[51]
*Moraxella *sp. C2, recombinant	10.0	7.3	80	7.5	NA	NA	[6]
*Pseudomonas *sp. 101	10.0	7.3	60	7	> 200	4.2 × 10^-4^	[6]

* NA – Data not available.

Plant enzymes are highly homologous (approximately 80%); the similarity between the bacterial and plant FDHs is ~50%.

## OBTAINMENT OF PLANT FORMATE DEHYDROGENASES

In plants, FDHs are localized in mitochondria; they therefore constitute a small part of all soluble proteins within a cell, and the isolation of an enzyme directly from plants is a labour-intensive and time-consuming procedure. Plant FDHs are mostly not very stable, which results in an appreciably significant inactivation of the enzyme during the extraction. Therefore, the specific activity of the resulting FDH specimens is much lower than one may expect ( *Table 2* ). The purified plant FDH was first obtained in 1951 from pea and French bean [13]. In 1983, FDH was extracted from soybean *G.max* in appreciably large amounts; this enabled the determination of the amino acid composition of plant formate dehydrogenase [57]. 

Since 1993 several cDNA of plant FDH have been cloned: in 1993 – from potato [21], in 1998 – from barley [8], in 2000 – from rice [41] and *A. thaliana* [26, 27]. Transgenic *A. thaliana* and tobacco plants expressing AthFDH were developed [28]; however, the yield of the enzyme was not very high. The expression of full size cDNA of potato FDH in *Escherichia coli * cells yielded insoluble inclusion bodies [21]. 

The active and soluble recombinant formate dehydrogenase from plants was first obtained in *E. coli * cells, [41]; however, the protein content was very low, approximately 0.01% of all soluble proteins within the cell. In our laboratory, we obtained *E. coli* strains, which are superproducers of active FDHs from *A. thaliana* (AthFDH) and soybean *G. max* (isoenzyme SoyFDH2) with an enzyme content attaining 40% of all soluble proteins in the cell [58] (the gene of soybean FDH was kindly provided by Professor N. Labrou from the Agricultural University of Athens (Greece); the FDH gene from *A. thaliana* , by Professor J. Markwell from the University of Nebraska (Lincoln, USA)). There is no system of transport to mitochondria in *E. coli * cells; therefore, in order to obtain the “natural enzyme”, we deleted the sequences encoding the signal peptide from the cDNA [58]. After the optimization of the cultivation conditions, the yield of recombinant FDH from *A. thaliana* and soybean *G. max * reached 500–600 mg/l of the medium [6]. A procedure was developed which enabled the obtaining of several hundred milligrams of homogenous FDH specimen via a single chromatographic stage per extraction run. Thus, all necessary conditions for the performance of systematic studies of FDHs from *A. thaliana* and soybean *G. max* were provided, including genetic engineering experiments and X-ray diffraction determination of the structure. The experiments were successfully carried out for the expression of FDH from *L. japonicas * in *E. coli * cells [10]. 

## KINETIC PROPERTIES OF PLANT FORMATE DEHYDROGENASE

*Table 2* summarizes the kinetic properties of natural and recombinant plant FDHs. The characteristics of the most thoroughly studied bacterial and yeast enzymes are provided for comparative purposes. Several major conclusions can be drawn from the data in *Table 2* : 

1. It is clearly visible that in case of FDH from *A. thaliana * the multistage extraction of the natural enzyme is accompanied by a considerable loss in activity. The specific activity of the specimens, even those obtained from transgenic plants, is several times lower than the activity of recombinant AthFDH expressed in *E. coli* cells. It is noteworthy that AthFDH belongs to stable FDHs. In terms of thermal stability, it is even superior to FDH from *Moraxella * sp. C2 and yeast *C. boidinii* ( *Table 3* ). It is obvious that the degree of inactivation of less stable, FDHs (in particular, in the case of SoyFDH) being extracted from natural sources will be higher. This fact should be taken into consideration when analysing formate dehydrogenase activity in plants. 

2. The specific activity of recombinant AthFDH and SoyFDH is comparable with that of formate dehydrogenases from microorganisms; although it is inferior to FDHs from methylotrophic bacteria and baker’s yeast. As previously mentioned, a partial inactivation of enzymes may occur during the extraction; therefore, calculation of the catalytic constant based on the values of specific activity and molecular weight may give underestimated *k*_cat_ values. This fact is of particular significance for SoyFDH, the thermal stability of which is much lower than that of other FDHs (see the thermal stability section below). Therefore, we developed a procedure for determining the concentration of active sites of recombinant SoyFDH based on the quenching of the intrinsic fluorescence of the enzyme as it is titrated with azide ion in the presence of coenzyme NAD ^+^ [64]. The azide ion is a strong competitive inhibitor of SoyFDH (K *I* = 3.6 × 10 ^-7^  M). Therefore, a linear dependence of quenching of FDH fluorescence on the azide concentration is observed under the conditions ensuring the equimolar binding of enzyme and inhibitor. The *k*_cat _ value determined from these experiments actually coincided with that calculated using the specific activity and molecular weight. The obtained data attest to the fact that despite the low thermal stability, SoyFDH extracted using the designed procedure is not inactivated. Today, this procedure is being actively used for determining the *k*_cat _ values of the mutant forms of SoyFDH. 

**Table 3:** Parameters of thermal inactivation of formate dehydrogenases from different sources

FDH source	Kinetics of thermal inactivation*		Differential scanning calorimetry* [72]		
	Δ*H*^≠^kJ/mol	Δ*S*^≠^, J/(mol·K)	*C*_p_, kJ/mol	*T*_m_,^o^C	*T*_1/2_,^o^C
*Pseudomonas sp. 101*	540 [6]	1320 [6]	2060	67.6	5.4
*Moraxella sp. C2*	NA**	NA**	1830	63.4	4.9
*Candida boidinii*	480 [77]	1250 [77]	1730	64.4	5.3
*Saccharomyces cerevisiae*	420 [67]	NA	820	46.4	3.2
*Arabidopsis thaliana*	490 [6]	1200 [6]	1330	64.9	5.9
*Glycine max*	370 [76]	860 [76]	820	57.1	7.5

* All measurements were carried out in the 0.1 M phosphate buffer, pH 7.0

** Data not available

3. Plant FDHs are characterized by significantly lower values of the Michaelis constants with respect to both formate and coenzyme NAD ^+^ in comparison with the bacterial and yeast enzymes. It is very important for the practical application of FDH. Today, recombinant FDHs from *Pseudomonas* sp. 101 and *C. boidinii* , whose chemical and thermal stability have been enhanced by protein engineering methods, are used for the regeneration of the reduced coenzyme (NADH or NADPH) in the synthesis of chiral compounds using dehydrogenases [6]. 

4. Almost all FDH that have been studied are highly specific to the coenzyme NAD ^+^ . Their catalytic activity in the reaction with NAD ^+^ is higher than that in the reaction with NADP ^+^ by a factor of 2500 (PseFDH) to 3 × 10 ^9^ . The exception is the recently described FDH from pathogenic bacteria *Burkholderia stabilis* , which is twenty-six-fold more efficient in the reaction with NADP ^+^ in comparison with NAD ^+^ [51]. It should be noted that the mutant PseFDH, whose coenzyme specificity changed from NAD ^+^ to NADP ^+^ , was obtained as early as in 1993 [6, 68]; this enzyme has been successfully used for NADPH regeneration [69, 70]. Having the lowest *К*_m _ value with respect to NADP ^+ ^ among all NAD ^+^ -specific wild-type FDHs, SoyFDH has a huge potential for obtaining the NADP ^+^ -specific enzyme [58] ( *Table 2* ). 

## THERMAL STABILITY OF PLANT FORMATE DEHYDROGENASES

Prior to obtaining recombinant AthFDH and SoyFDH in *E. coli * cells, no systematic studies on the thermal stability of plant FDHs were performed. According to [59], the values of К _M_ with respect to formate and NAD ^+^ decrease after the incubation of transgenic AthFDH for 5 min at 60°С; however, the specific activity value decreased by 13 times. We performed systematic studies of the thermal stability of recombinant AthFDH and SoyFDH using two approaches: determining the kinetics of thermal inactivation and the differential scanning calorimetry [71, 72]. It was revealed that the thermal inactivation of plant FDHs occurred via a monomolecular mechanism, similar to the FDHs from bacteria and yeasts. Time dependences of decrease in the activity of AthFDH and SoyFDH are described by the kinetics of the first-order reaction, the observed constant of inactivation rate was independent of enzyme concentration. The thermal stabilities of AthFDH and SoyFDH strongly differ. AthFDH lost 50% of its activity after 20 min at 59.5°С, whereas SoyFDH lost 50% of its activity at 52.8°С. The difference by almost 7°С corresponds to a difference in the inactivation rate constants of more than 1000 times. AthFDH is actually inferior in terms of its thermal stability only to FDH from *Pseudomonas * sp. 101 (63.0°С, the most stable FDH among the known ones) [6] and *Staphylococcus aureus* (62.0°С) and is superior to all known microbial formate dehydrogenases. In contrast, SoyFDH is inferior in terms of stability to all known FDHs, with the exception of the enzyme from baker’s yeast that is characterized by another inactivation mechanism. The temperature dependences of the rate constants of inactivation of AthFDH and SoyFDH are described by the transition state theory. The calculated values of activation enthalpy Δ *H ^≠^* and activation entropy Δ *S ^≠ ^* are listed in *Table 3. * It can be noticed that thermal stability of formate dehydrogenases correlates well with the Δ *H ^≠^* and Δ *S ^≠ ^* values. The highest values are typical of the most stable PseFDH, whereas the lowest ones refer to SoyFDH. 

**Fig. 4:**
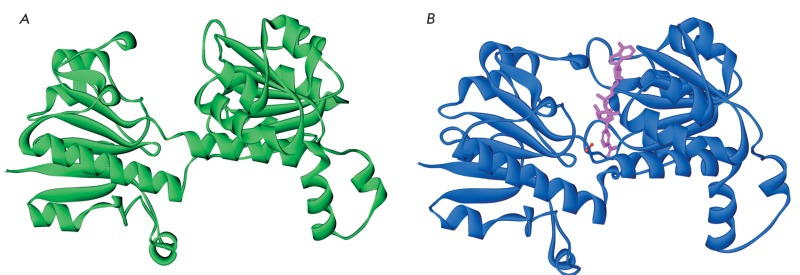
The structures of apo-(A) and holo-(B) forms of FDH from *A. thaliana.* Figures were obtained using PDB structures 3JTM and 3N7U, respectively. In the structure of the holo-form, the molecules of NAD ^+^ and azide ion are highlighted in magenta and red, respectively.

The results of experiments studying the thermal stability of plant FDHs using differential scanning calorimetry are in close agreement with the data of inactivation kinetics. In *Table 3* the values of the temperature and heat of the phase transition are shown. All investigated FDHs are characterized by their high cooperativity of the denaturation process. PseFDH is characterized by the highest melting point, whereas this value is lower by a factor of 2.5 for SoyFDH. The melting point of AthFDH is higher than that of CboFDH and MorFDH. 

Experiments on enhancing the thermal stability of recombinant SoyFDH using genetic engineering techniques are currently being performed.

## CHEMICAL STABILITY OF PLANT FORMATE DEHYDROGENASES

FDH has been actively used in the process of synthesizing optically active compounds catalyzed by dehydrogenases. In these processes, the operational stability (the operating time of an enzyme) plays the greatest role. Under conditions of biocatalytic process, the inactivation of FDH is associated with either oxidation with oxygen or chemical modification of sulfhydryl groups of the enzyme. The operational stability of PseFDH and CboFDH was enhanced by site-directed mutagenesis of two Cys residues [6, 65]. As previously mentioned, the synthesis of FDHs in plants increased under various stress factors. The concentration of active forms of oxygen (superoxide radicals, hydrogen peroxide, etc.) is likely to increase in a cell under the same conditions. It should be expected that in order to ensure high activity under stress, plant FDHs should be far more resistant to the action of these agents than formate dehydrogenases functioning under non-stress conditions. In order to verify this hypothesis, the kinetics of inactivation of recombinant AthFDH, SoyFDH, PseFDH, FDH from wild-type *S. aureus* (SauFDH) and three mutant PseFDH, where one or two Cys residues were replaced, under the action of hydrogen peroxide was studied. FDH from *S. aureus * is a stress protein as well, since the biosynthesis of this enzyme increases by 20 times when staphylococci pass from planktonic growth to biofilm formation [73]. It was revealed that the inactivation rate of AthFDH and SoyFDH under the action of Н _2_ О _2_ is virtually equal and is 18 times lower than that in wild-type PseFDH. Under the same conditions, the stability of SauFDH was sixfold higher than that in plant enzymes [74]. PseFDH with the stability identical to that of SoyFDH was successfully obtained only after double Cys145Ser/Cys255/Ala replacement. It was thus demonstrated that plant and bacterial FDHs synthesized under stress impact indeed possess a higher chemical stability than formate dehydrogenases, whose synthesis is induced by other factors. Moreover, studying the thermal inactivation of formate dehydrogenase in the presence of hydrogen peroxide can be used to comparatively assess the * in vivo* chemical stability of FDHs. 

## STRUCTURAL STUDIES OF PLANT FORMATE DEHYDROGENASES

FDH has not been well studied from a structural perspective. Until recently, only the FDH structures from three sources, namely, bacteria *Pseudomonas* sp. 101 and *Moraxella*  sp. C2 (unbound enzyme, and the enzyme–NAD ^+^ –azide ternary complex) and *C. boidinii* yeast (structures of two mutant forms of an apoenzyme) were deposited into the protein data bank (PDB). In case of formate dehydrohenases, free enzyme is in its «open» conformation. When the FDH–NAD ^+^ –azide ternary complex is formed (its structure being considered similar to that of the enzyme in the transition state), a considerable compacting of the protein globule occurs, and FDH is transformed into the «closed» state. The development of a highly efficient system of expression of AthFDH and SoyFDH *in E. coli* cells enabled the transition to their crystallization and identification of their structure., Nowadays AthFDH structures both in open and closed conformations (3NAQ and 3N7U, with resolution of 1.7 and 2.0 Å, respectively) have been identified. Enzyme crystals were produced in space [75] in order to obtain AthFDH structure in open conformation with higher resolution (3JTM, 1.3 Å), *Fig.*  4 shows the structures of apo- and holoFDHs (the open and closed conformations, respectively). A more detailed analysis of these structures will be provided in individual articles. The crystals of the SoyFDH–NAD ^+^ –azide ternary complex of FDH from soybean *G. max* have been obtained both on the Earth and in space. The structures of these complexes were identified with resolution of 1.9 Å; the deposition of these structures into the PDB data bank is in progress. 

## CONCLUSIONS

Both our own and the published data attest to the fact that plant FDHs are extremely significant, in particular when responding to stress factors. Biosynthesis regulation and the physiological role of FDHs are diverse and complex; they have not been completely elucidated thus far. The systematic investigation of the structure and function of these enzymes is still in its infancy. The results of these studies are of significant interest to fundamental science and are of great practical importance. Production of mutant forms of FDH with a high activity opens a new approach for the design of plants with an enhanced resistance to unfavourable factors. More active mutant enzymes will supply the cell with the energy required to more efficiently overcome unfavourable effects of stress with the same expression level of FDH. Soybean FDH is also considered to be an exceptionally promising FDH for the design of a highly efficient biocatalyst for NAD(P)H regeneration in the synthesis of optically active compounds using dehydrogenases. The natural enzyme is notable for its high operational stability and has one of the lowest values of the Michaelis constant with respect both to NAD ^+^ and formate among all of the known FDHs. However, in order to practically implement SoyFDH, its catalytic activity and thermal stability need to be enhanced. We are currently performing active research in this direction. 

## Abbreviations

FDH: formate dehydrogenase
PseFDH, CboFDH: formate dehydrogenases from bacteria*Pseudomonas*sp
101 and yeast*Candida boidinii*, respectively*;*SoyFDH,**AthFDH: plant formate dehydrogenases from soybean*Glycine max*and*Arabidopsis thaliana*, respectively
